# Mitigating Effect of Matricin Against Benzo(a)pyrene-induced Lung Carcinogenesis in Experimental Mice Model

**DOI:** 10.2174/0113862073273177231130094833

**Published:** 2024-01-09

**Authors:** Guang Yang, Huining Liu, Siwei Xu, Ziqiang Tian

**Affiliations:** 1 Department of Thoracic Surgery, The First Hospital of Hebei Medical University, Shijiazhuang, Hebei Province, 050000, China;; 2 Department of Thoracic Surgery, The Fourth Hospital of Hebei Medical University, Shijiazhuang, Hebei Province, 050011, China

**Keywords:** Apoptosis, immunoglobulin, tumor, antioxidants, inflammation, matricin, benzo(a)pyrene [B(a)P]-induced lung cancer

## Abstract

**Background:**

Lung cancer is a life-threatening disease that is still prevalent worldwide. This study aims to evaluate the effects of matricin, a sesquiterpene, on the carcinogenic agent benzo(a)pyrene [B(a)P]-induced lung cancer in Swiss albino mice.

**Methods:**

Lung cancer was induced by oral administration of B(a)P at 50 mg/kg b. wt. in model Swiss-albino mice (group II) as well in experimental group III, and treated with matricin (100 mg/kg b. wt.) in group III. Upon completion of treatment for 18 weeks, the changes in body weight, tumor formation, enzymatic and non-enzymatic antioxidant levels (GSH, SOD, GPx, GR, QR, CAT), lipid peroxidation (LPO) level, pro-inflammatory cytokines (TNF-α, IL-6, IL-1β), immunoglobulin levels (IgG, IgM), apoptosis markers (Bax, Bcl-xL), tumor markers (carcinoembryogenic antigen (CEA), neuron-specific enolase (NSE)), and histopathological (H&E) alterations were determined.

**Results:**

The results indicate that B(a)P caused a significant increase of tumor formation in the lungs, increased tumor markers and inflammatory cytokines in serum, and depletion of enzymatic/non-enzymatic antioxidants and immunoglobulins, compared to the untreated control group. Matricin treatment significantly reversed the changes caused by B(a)P as evidenced by the biochemical and histopathological assays.

**Conclusion:**

The changes caused by matricin clearly indicate the cancer-preventive effects of matricin against B(a)P-induced lung cancer in animal models, which can be attributed to the antioxidant activity, immunomodulation, and mitigation of the NF-kβ pathway.

## 1 INTRODUCTION

Cancer is the second most death-causing disease in the world and lung cancer has been prevalent among the other types of cancer in terms of fatalities in the past years. The role of cancer-causing agents and the pathogenesis of lung cancer are widely studied and there are efforts to map the mechanisms involved in the progression of cancer [1]. Lung carcinogenesis in animal models for preclinical study is well established using benzo(a)pyrene [B(a)P], a carcinogen found in the smoke of vehicle exhaust and cigarettes [2, 3]. The pro-carcinogenic B(a)P is biotransformed by cytochrome P450 and subsequent enzymes into a highly reactive electrophilic metabolite B(a)P‐7,8‐diol‐9,10‐epoxide. These reactive metabolites attack the healthy lung cells, and target bulk DNA thus forming DNA adducts at guanine residues and triggering protein expression for the prevention of DNA repair, and apoptosis, and intercepting other cell proliferation activities. Other intermediate metabolites of B(a)P such as o-quinones cause an upsurge in free radical formations like reactive oxygen species (ROS) and reactive nitrogen species (RNS) upon undergoing redox cycling with semiquinone derivatives [4]. Elevated levels of ROS lead to severe DNA damage and oxidative stress, hence serving as the pathogenesis for lung carcinogenesis to develop.

Although the current clinical approach uses chemotherapy and radiotherapy for lung cancer treatment, the adverse effects are mostly irreversible. Adverse conditions such as neutropenia and leukopenia often lead to increased morbidity and motility among lung cancer patients [5]. It is also learned that immunoglobulins are produced in cancer patients and inadvertently support the progression of the disease by creating a defense mechanism for tumor cells. Therefore, a potential drug should be discovered for the prevention of cancer progression, decrease chemoresistance, and effectively kill the tumors with minimal or no side effects to the patient. There are plenty of pre-clinical and few clinical studies on new drug candidates to treat lung cancer. Recently, natural products have garnered interest among researchers as these products are safe for human consumption and have been a rich source of compounds with pharmacological relevance [6,7]. Naturally occurring bioactive metabolites have antioxidant properties that could possibly intercept the pathogenesis of lung cancer mediated by free radicals. The amide functional groups, specifically benzamide derivatives are promising building blocks for any bioactive compounds whereby they play important roles in the biochemical processing in nature. Chemical synthesis of drugs using these ligands is much preferred as they could exert the desired anticancer properties [8]. Matricin is a bioactive sesquiterpene that is found in *Matricaria recutita* L., also known as the chamomile plant. Pharmacological properties of matricin upon pre-clinical evaluation details into antioxidant, anti-inflammatory, anti-microbial, and anti-cancer properties on several *in-vitro* experiments [9, 10]. A recent study on the effect of matricin on human non-small cell lung cancer H1299 reportedly induced apoptosis and regulated the MAPK pathway [11]. To our knowledge, this is the first report on the effect of matricin in B(a)P-induced lung carcinogenesis. Therefore, this study aims to evaluate the anti-cancer effect of matricin against B(a)P-induced lung carcinogenesis in an experimental mice model.

## 2 MATERIALS AND METHODS

### 2.1 Chemicals

Matricin and B(a)P were purchased from Sigma, St. Louis, US. All other chemicals and reagents purchased were of the highest grade experimental quality.

### 2.2 Animals

Male Swiss-albino mice (7-8 weeks old, 25-30 g) were obtained from the institutional animal house facility and were treated in a humane manner following the Institutional Animal Ethical Committee guidelines (No.: IACUC-Hebmu-20230121) of Hebei Medical University, China and also following STROBE guidelines. Animals were acclimatized a week prior experiment and was allowed access to standard food pellet and water *ad libitum*. Animals were caged in clean and dry polypropylene cages, with a 12 h light / 12 h dark cycle and humidity of 25 ± 2°C.

### 2.3 Experimental Protocol

Experimental grouping was determined based on the method of Velli *et al*. [12]. Dose selection for matricin was done based on preliminary studies and past *in-vitro* model studies on matricin [9]. Twenty-four mice were randomly separated into four groups (n = 6):

Group I: Untreated control, orally received corn oil for 18 weeks.

Group II: Model group, orally received B(a)P (50 mg/kg b.wt.) in corn oil (1:1) twice a week for four consecutive weeks (week 2-5), followed by corn oil only until week 18.

Group III: Experimental group, orally received B(a)P (50 mg/kg b.wt.) in corn oil (1:1) twice a week for four consecutive weeks (week 2-5), followed by matricin (100 mg/kg b.wt.) daily from week 12 to 18.

Group IV: Drug control, orally received matricin (100 mg/kg b.wt.) daily for 18 weeks.

All animals were sacrificed 24 h after the last treatment under anesthesia (ketamine 90 mg/kg b.wt.) followed by cervical dislocation for lung and blood collection. Lung tissues were removed, tumor incidence and weight of lungs were measured. A section of the lung was stored in 10% neutralized formalin for histopathological analysis. Lung tissues were homogenized in 25 mM Tris-HCl buffer (pH 7.4), centrifuged (4°C) at 2 000 rpm for 5 min to collect nuclear debris, and the supernatant obtained was further centrifuged at 12 000 rpm for 20 min to collect post-mitochondrial supernatant for biochemical assays. Blood samples were centrifuged (4°C) at 3,000 rpm for 15 min to collect serum for further biochemical analysis. Protein determination was performed using the Bradford method.

### 2.4 Biochemical Assays

Biochemical analyses were performed following the method of Shahid *et al*. [13]. Briefly, reduced glutathione (GSH), glutathione peroxidase (GPx), glutathione reductase (GR), superoxide dismutase (SOD), quinone reductase (QR), and catalase (CAT) levels in post-mitochondrial supernatants were determined using standard diagnostic assay kits (Sigma, St. Louis, US), following the protocols described by Gong *et al* [14]. Lipid peroxidation (LPO) in post-mitochondrial supernatants was also measured using a standard colorimetric assay kit (Sigma, St. Louis, US) to quantify the malondialdehyde (MDA) formations according to the method of Gnanaraj *et al.* [15].

### 2.5 ELISA Assays

Serum levels of the pro-inflammatory cytokines (TNF-α, IL-1β, IL-6), serum immunoglobulin levels (IgG, IgM), and apoptosis markers in tissue (Bax, Bcl-xL) were estimated using ELISA assay kits (Abcam, Cambridge, UK) according to the manufacturer’s protocols. Serum levels of tumor marker enzymes CEA and NSE were determined using ELISA assay kits (Cell Signaling Technology, Beverly, US) following the manufacturer’s protocols.

### 2.6 Lung Histopathological Analysis

Histopathological analysis of lung tissues was performed on the mid-lobe of the lung portion stored in 10% neutralized formalin. The tissues were processed and dehydrated using ethanol gradients, embedded in paraffin, and sectioned to 5 µm thick, then de-paraffinized with ethanol and xylene, and finally stained with hematoxylin and eosin (H&E). The H&E stained tissues were visualized under a microscope with photographic facilities. The pathological changes were verified by a clinical pathologist blinded to the experimental grouping.

### 2.7 Statistical Analysis

Statistical analysis was performed on data for all groups using SPSS 22.0, Chicago, US, and presented as mean ± standard deviation. Raw data were tested for normality and subjected to analysis of variance (one-way ANOVA) followed by the Tukey-Kramer multiple comparisons test. All differences that had ap-value less than 0.05 were considered statistically significant [16].

## 3 RESULTS

### 3.1 Effect of Matricin on Body Weight and Tumor Formations

**Fig. (1):**
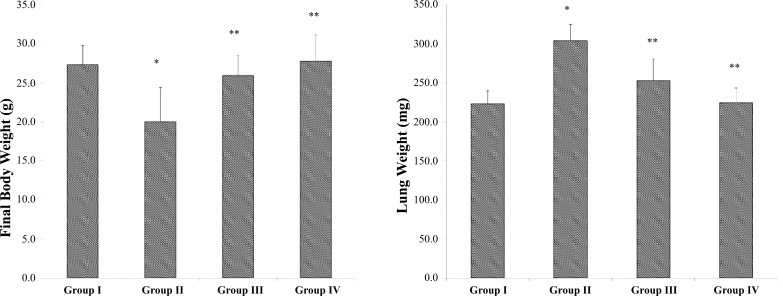
Matricin affects the body and lung weight of B(a)P-induced experimental animals. All values represent the mean ± SD of six animals (n = 6). Statistical analysis was performed by one-way ANOVA followed by the Tukey-Kramer multiple comparisons test. Single asterisk (*) represents significant changes from normal control group I (*p* < 0.05), and double asterisks (**) represent significant difference from B(a)P model group II (*p* < 0.01).

The body weight of animals was measured and B(a)P treated model group II had lower final body weight compared to the normal control group I. Matricin-treated group III showed improved body weight compared to the B(a)P alone treated group II and there were no significant differences between the matricin control group IV and normal control group I (Fig. **1**). The lung weight of B(a)P treated model group II was significantly higher than all other groups, whereas the lung weight of matricin-treated group III was lower than that of model group II (Fig. **1**). Tumor formations were calculated in the lungs of animals and was found that tumor incidence per mice in the B(a)P treated model group was prominent (6.3 ± 0.8) and matricin-treated group III showed minimal tumor formations per mice (2.7 ± 0.9). Both control groups did not show any tumor formations.

### 3.2 Effect of Matricin on Antioxidant Defense System

**Fig. (2):**
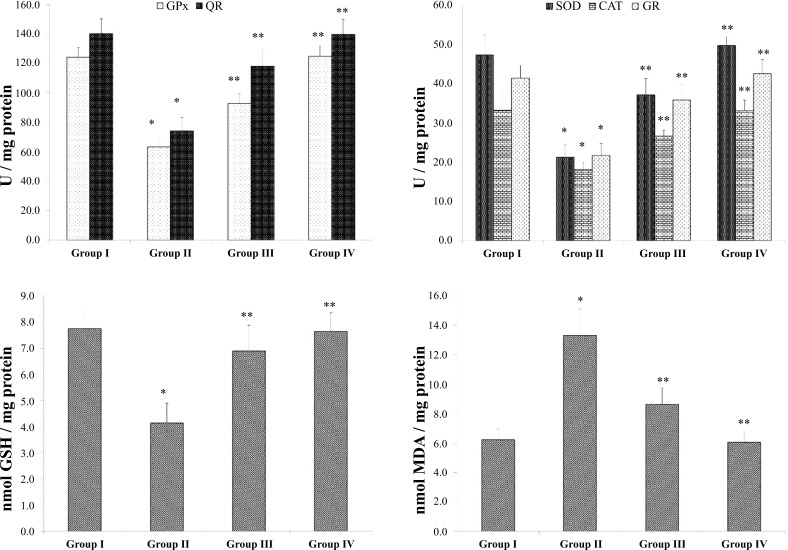
Matricin affects the levels of the non-enzymatic (GSH), enzymatic antioxidants (SOD, GPx, GR, CAT, QR), and lipid peroxidation (MDA formation). All values represent the mean ± SD of six animals (n = 6). Statistical analysis was performed by one-way ANOVA followed by the Tukey-Kramer multiple comparisons test. Single asterisk (*) represents significant changes from normal control group I (*p* < 0.05), and double asterisks (**) represent significant difference from B(a)P model group II (*p* < 0.01).

The levels of glutathione-based antioxidant defense system (GSH, GPx, GR) and enzymatic antioxidants (SOD, QR, CAT) were ultimately lowered in the B(a)P model group II compared to the normal group I, but matricin-treated group III increased the levels of the non-enzymatic and enzymatic antioxidants compared to the B(a)P model group II (Fig. **2**). Matricin control group IV showed equal levels of the antioxidant defense markers, with the control group I. Lipid peroxidation levels were also measured and were significantly increased by 114% in B(a)P model group II compared to normal group I. Matricin-treated group III showed a reduction in the MDA formation by 76% compared to B(a)P model group II (Fig. **2**). Drug control group IV exhibited minimal MDA formation similar to the normal group I.

### 3.3 Effect of Matricin on Pro-inflammatory Cytokines and Apoptosis Markers

**Fig. (3):**
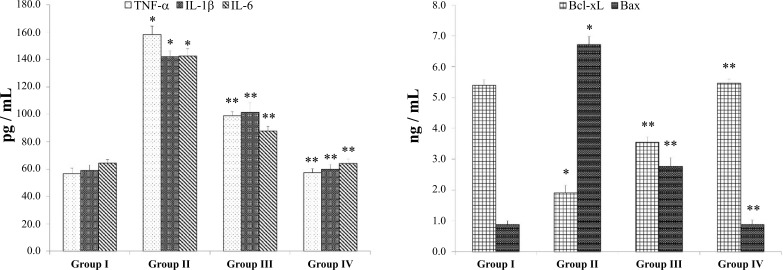
Matricin attenuates the levels of pro-inflammatory cytokines (TNF-α, IL-1β, IL-6) and apoptosis markers (Bax, Bcl-xL). All values represent the mean ± SD of six animals (n = 6). Statistical analysis was performed by one-way ANOVA followed by the Tukey-Kramer multiple comparisons test. Single asterisk (*) represents significant changes from normal control group I (*p* < 0.05), and double asterisks (**) represent significant difference from B(a)P model group II (*p* < 0.01).

The level of pro-inflammatory cytokines (TNF-α, IL-1β, IL-6) were significantly increased in the B(a)P model group II compared to the normal group I, in contrast, matricin treated group III decreased the levels the pro-inflammatory cytokines compared to model group II (Fig. **3**). Apoptosis marker Bax was significantly upregulated in B(a)P model group II compared to the normal group I, whereas Bcl-xL was downregulated in model group II compared to group I. Matricin-treated group III significantly reversed the expression of apoptosis markers by lowering Bax and increasing Bcl-xL to normal levels (Fig. **3**). Matricin control group IV did not show any changes in the levels of pro-inflammatory cytokines and apoptosis markers similar to group I.

### 3.4 Effect of Matricin on Immunoglobulin IgG and IgM

**Fig. (4):**
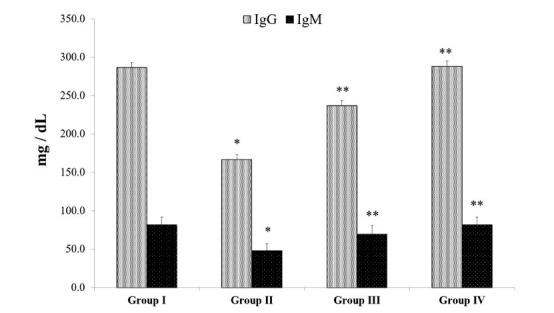
Modulatory effects of matricin on immunoglobulin levels of IgG and IgM in B(a)P-induced experimental animals. All values represent mean ± SD of six animals (n = 6). Statistical analysis was performed by one-way ANOVA followed by the Tukey-Kramer multiple comparisons test. Single asterisk (*) represents significant changes from normal control group I (*p* < 0.05), and double asterisks (**) represent significant difference from B(a)P model group II (*p* < 0.01).

Immunoglobulin IgG and IgM levels were measured in the serum of animals. The levels of IgG and IgM were decreased significantly in B(a)P model group II compared to control group I whereas matricin treatment in group III significantly reversed the effect by increasing the levels of IgG and IgM compared to B(a)P model group II (Fig. **4**). The levels of IgG and IgM in matricin control group IV were normal, similar to control group I.

### 3.5 Effect of Matricin on Tumor Markers CEA and NSE

**Fig. (5):**
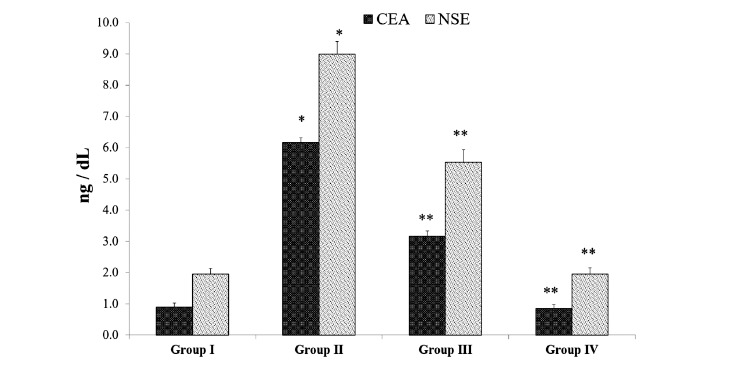
Matricin effectively suppresses tumor markers (CEA and NSE) in B(a)P-induced experimental animals. All values represent the mean ± SD of six animals (n = 6). Statistical analysis was performed by one-way ANOVA followed by the Tukey-Kramer multiple comparisons test. Single asterisk (*) represents significant changes from normal control group I (*p* < 0.05), and double asterisks (**) represent significant difference from B(a)P model group II (*p* < 0.01).

Tumor formation was evidenced visually on B(a)P model group II, hence the tumor markers CEA and NSE were also shown to be elevated in group II by 438% and 389% respectively compared to the normal control group I. The levels of CEA and NSE were reduced significantly by matricin treatment in group III by 66% and 52% respectively compared to B(a)P model group II (Fig. **5**). Matricin control group IV exhibited comparable levels of CEA and NSE with control group I.

### 3.6 Histopathological Changes in the Lung by B(a)P and Matricin

**Fig. (6):**
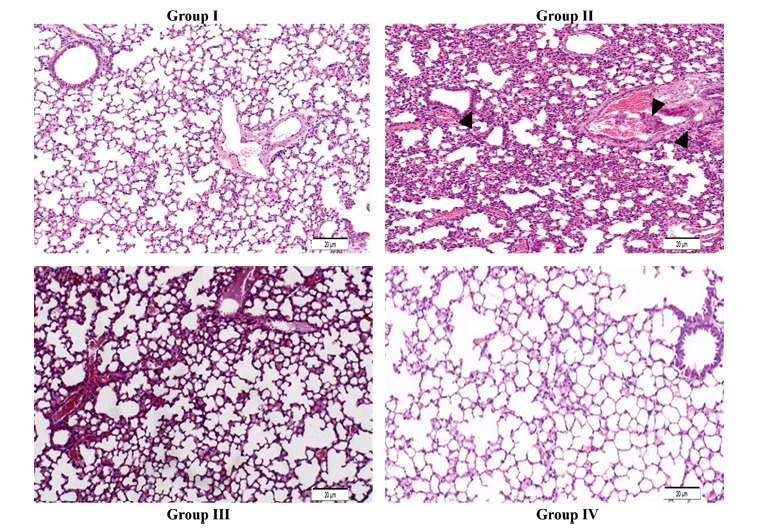
Effect of matricin on pathological alterations in the lung of B(a)P-induced experimental animals. H&E staining of lung histopathology shows normal control group I as normal lung architecture whereas the B(a)P model group II shows excessive hyperchromatic nuclei, wide proliferation of alveolar epithelium and appearance of pulmonary carcinoma characterized by tumor nodule, as depicted by the arrows. Matricin treatment in group III shows an almost normal architecture of the lung with minimal changes. Matricin alone treated group IV exhibited normal lung architecture comparable to group I. Magnification was at 100×.

H&E staining on the normal control group I exhibited healthy lung cells with normal architecture and uniform nuclei. In contrast, B(a)P model group II exhibited damaged alveolar architecture, irregular nuclei with deformity, and infiltration of inflammatory cells. Matricin-treated group III exhibited protective effects with only minor deformity of alveolar architecture (Fig. **6**). Matricin control group IV showed healthy architecture of the lung cells similar to normal control group I.

## 4 DISCUSSION

Body weight and lung index of B(a)P-induced animals with lung cancer were abnormal following the pattern displayed in past studies [12, 17]. The body weight of animals in the B(a)P-induced model group substantially dropped whereas the lung weight was enlarged compared to the normal control group. The enlarged lungs are signs of tumor formation that indicates neoplasm reaction such as uncontrolled proliferation of cells, infiltration of inflammatory mediators, and enlargement of nodules. Tumor formation in B(a)P-induced animals was prominent but matricin suppressed the tumor incidence. Plant secondary metabolites are known for their anti-cancer properties and anti-inflammatory effects [4]. Matricin prevented the enlargement of lungs and retained body weights of B(a)P-induced animals, expressing the potential of the natural sesquiterpene in inhibiting cancer progression.

B(a)P is a strong carcinogen with the capability to trigger enormous free radical formations. LPO is a result of free radicals attacking the lipid membrane of cells, to form harmful adducts [18]. LPO levels were significantly high in the B(a)P-induced model group, measured by MDA formations but matricin prevented MDA formation suggesting the alteration of LPO. The lung tissues of the B(a)P-induced model group were considered to have been in a state of oxidative stress since the enzymatic and non-enzymatic antioxidants were almost depleted. SOD, GPx, GR, and CAT are enzymes that convert free radicals into harmless products to be excreted out of the body. Superoxide anions and free radicals are converted to hydrogen peroxide by SOD, whereas GPx and CAT converts the hydrogen peroxide to water [13]. GR recycles oxidized glutathione disulfide (GSSG) back to its reduced form (GSH). QR on the other hand is a phase II enzyme that detoxifies the tissues from the harmful electrophilic quinone radicals. These enzymes are clustered together in fighting oxidative stress. The carcinogenic effect of B(a)P causes the activities of the enzymatic and non-enzymatic antioxidants to be weakened hence leading to mutagenicity and cancerous effects to the cells. Matricin as a natural antioxidant was able to scavenge the free radicals formed by B(a)P and prevent oxidative stress. The activities of SOD, GPx, GR, CAT, QR, and GSH were significantly replenished by matricin. Similar results were reported in previous studies involving B(a)P and natural antioxidant compounds, suggesting the anti-cancer effects of the bioactive compounds [19, 20].

Oxidative stress is closely associated with cancer progression and treatment. An inflammatory reaction is triggered by oxidative stress since TNF-α is a pro-inflammatory cytokine produced by macrophages that regulates the tumor cell necrosis, inflammation, and immune response and these macrophages are usually stimulated under oxidative stress [21]. The pro-inflammatory cytokines TNF-α, IL-1β, and IL-6 were highly expressed in the B(a)P-induced model group similar to previous studies [12,13]. These pro-inflammatory cytokines are known to trigger a cascade of immunomodulatory reactions through the activation of the nuclear factor-kβ (NF-kβ) pathway responsible for the oxidative stress mechanism [7]. Immunomodulatory reactions due to B(a)P-induced carcinogenesis were evidenced by the suppression of antibodies IgG and IgM, a pattern exhibited by cancer patients with compromised humoral immunity [20]. The anti-inflammatory and immunomodulatory effect of matricin was proved as the pro-inflammatory cytokines were suppressed and antibodies IgG and IgM were restored to normal levels compared to the model group, postulating the efficiency of matricin in curbing oxidative stress and immune reactions in carcinogenesis. Apoptosis serves as an important activity in the prevention of cancer. The apoptosis activity is also related to oxidative stress whereby apoptosis proteins such as the Bcl-2 family regulate cancer cells [22, 23]. Bcl-xL, a transmembrane protein in mitochondria has an anti-apoptotic effect, as was found to be suppressed in B(a)P-induced model group, indicating the presence of cancer cell formation, whereas Bax, a pro-apoptotic protein was highly expressed in the same group. Matricin reversed the effects of these apoptotic markers, supporting the effectiveness against B(a)P-induced cancer progression.

The indication of tumor formation is validated by the detection of markers such as tumor-related glycoprotein, CEA which is an oncofetal antigen commonly considered as an early indicator. CEA is mostly expressed in healthy cells adjacent to malignant tumors that may further promote metastasis [19]. Augmented levels of CEA in B(a)P-induced animals indicated the formation of tumors, similarly reported by past studies [13,19]. NSE is a tumor marker that indicates the malignancy loads in lung cancer, through measuring energy intervention at serum levels hence serving as a useful marker for the diagnosis of cancer [13]. Augmented levels of NSE in B(a)P-induced animals also supported the evidence of tumor formations. Matricin significantly decreased the levels of both CEA and NSE in B(a)P-induced animals thus showing the suppression of tumor formation and inhibition of metastasis. Histopathological events clearly evidenced the tumor formations and changes in B(a)P-induced lung carcinogenesis that were remarkably altered by matricin. The histopathological changes support the biochemical and immunological findings, suggesting matricin with significant anti-cancer properties. The limitation of this study is the lengthy duration of the experiment that requires constant monitoring of the mice and the amount of matricin required for the treatment. Moreover, this model of preclinical study can only be used for the screening of potential bioactive compounds but not for mimicking human lung cancer.

## CONCLUSION

In conclusion, this study proved the efficiency of matricin in mitigating lung carcinogenesis induced by B(a)P in mice model through prevention of tumor formation, reduction of MDA formation, elevation of antioxidant enzymes SOD, QR, CAT, GPx, GR, GSH, reduction of inflammation (TNF-α, IL-6, IL-1β), reversal of apoptosis (Bax, Bcl-xL), immunomodulation of IgG and IgM, suppression of tumor markers (CEA, NSE) and prevention of pathological alterations. The mechanism involved in the attenuation of B(a)P-induced lung cancer by matricin is attributed to oxidative stress prevention, immunomodulation, and apoptosis regulation controlled by the NF-kβ pathway. This study contributes to the anti-cancer effects of matricin that can be further evaluated on different *in-vivo* cancer models to understand the underlying molecular mechanisms and pathways prior to consideration for clinical studies.

## Data Availability

The data and supportive information are available within the article.

## LIST OF ABBREVIATIONS

B(a)P: Benzo(a)Pyrene
CEA: Carcinoembryogenic Antigen
GPx: Glutathione Peroxidase
GR: Glutathione Reductase
GSH: Glutathione
LPO: Lipid Peroxidation
LPO: Lipid Peroxidation
MDA: Malondialdehyde
NSE: Neuron-Specific Enolase
QR: Quinone Reductase
RNS: Reactive Nitrogen Species
ROS: Reactive Oxygen Species
SOD: Superoxide Dismutase

## STANDARDS OF REPORTING

STROBE vet guidelines were followed.
